# Opiates in Extensive Burns

**Published:** 1845-06

**Authors:** H. Rowe


*Opiates in Extensive Burns.* By H. Rowe, M. D.—It is said that full doses of opiates in burns are followed by happy results; allow me to bear testimony to such facts. I have, for ten years and more, invariably used them in extensive burns, with unvarying success; and I remember, more particularly, one case that occurred to me while I was resident medical officer of the Demerara Seaman's Hospital. It was a boy, about 16 or 17 years of age, who was frightfully burned, the left arm, side, and abdomen, presenting a shocking appearance, and the nervous system, in proportion, violently affected (which is the real cause of deaths from burns). Opiates in *full* doses, mixed with effervescing draughts, and repeated after the effects had left, with protein dresssing of linseed oil and lime water in thick layers (which could be easily applied and kept on after the opiate had taken effect), with the *secundem artem* treatment aftewards, gave me the great gratification of seeing him leave the hospital well and hearty, which effect I must certainly attribute to the immediate use of opiates.—*Ibid.*

